# Influenza and Ultraviolet Germicidal Irradiation

**DOI:** 10.1186/1743-422X-5-149

**Published:** 2008-12-16

**Authors:** Lewis J Radonovich, Richard A Martinello, Michael Hodgson, Donald K Milton, Edward A Nardell

**Affiliations:** 1 Biosecurity Programs, Office of Program Development, North Florida/South Georgia Veterans Health System, University of Floirda, Florida, USA; 2 VA Connecticut Healthcare System, West Haven, CT, Yale School of Medicine, New Haven, CT, USA; 3 Veterans Health Administration, Washington, DC, USA; 4 University of Massachusetts Lowell, Harvard School of Public Health, Boston, MA, USA; 5 Harvard Medical School and School of Public Health, Brigham and Women's Hospital, Division of Global Health Equity, Boston, MA, USA

To The Editor:

In the February 25th issue of *Virology Journal*, Cannell and colleagues propose that Vitamin D is intimately associated with the incidence of human influenza infection to the extent that it explains numerous previously misunderstood manifestations of the disease and the behavior of epidemics [1]. Although a novel and intriguing hypothesis, it contains multiple incorrect assertions. Regarding the 1957 Livermore, California influenza study [2], Cannell and colleagues assert,

"Maclean's [sic] description of the Livermore hospital's procedures is inadequate to know if patients were being directly irradiated, thus triggering vitamin D production in their skin. However careful inspection of another 1957 publication about a similarly irradiated Baltimore VA hospital – co-authored by McLean – is illuminating [3]. The Baltimore hospital wing apparently used a similar irradiation set-up with 'standard ultraviolet fixtures.' (p. 421) illustrations clearly show – despite text stating that only upper air was irradiated – that the rooms and hallways were all equipped with UV lights that either shone directly or indirectly on patients ... [which] would have significantly raised the 25(OH)D levels of the irradiated, and relatively influenza free, patients. "

Represented in this assertion are multiple key misconceptions about air disinfection using ultraviolet germicidal irradiation (UVGI) that need to be rectified.

First, McLean clearly identifies the location of the air disinfection as the upper-room, as was the practice at the time. In the text under Figure 4 (p. 37) he describes the UV treated ward of the Livermore facility as sustaining "disinfection of the upper air of all rooms and corridors [2]." Investigators at the time were well aware of the need to confine UV to the upper room for occupant safety [4,5]. In addition, the UV fixtures in the Baltimore hospital wing, in fact, did *not *directly irradiate the buildings occupants [3]. The fixtures directed the UV energy horizontally and vertically upward, thereby minimizing UV exposure in the occupied space below. It is true that a small fraction of the UVC emitted from the fixture was reflected back down toward the occupied space; however, since the UV fixtures were located in the upper-room (*e.g*., above seven feet), the amount of UV reaching the occupants was necessarily less than 6 mJ/cm^2 ^(the Threshold Limit Value or TLV) over 8 hours of exposure to prevent eye irritation [6-9]. In contrast, two hours peak exposure to sunlight in summertime can deliver approximately 740 mJ/cm^2 ^of much more penetrating longer wavelength UV [10].

Second, even if the subjects' skin was significantly exposed to UVGI, little or no vitamin D production would have been stimulated. The UVGI sources used in the 1950s [11] (and today [12,13]) for air disinfection in the healthcare setting were low-pressure mercury vapor lamps which emit short wave ultraviolet (almost entirely 253.7 nm) irradiation (UVC range). The amount of UVC that crosses the outer dead layer of the skin (stratum corneum) [14] and the amount of vitamin D synthesis that occurs from UVC exposure [15] are clinically negligible. In contrast, UV in sunlight is entirely longer wavelength UV with many times the penetration to reach vitamin D generating cells. From a practical standpoint, doses of UVC required to alter 7-dehydrocholesterol metabolism would cause substantial eye irritation (photokeratoconjunctivitis). Such adverse events were rarely if ever reported.

Third and finally, Cannell *et al *make the case that school-based UVGI studies showed no benefit, citing three studies widely acknowledged to be flawed in design [16] that showed no effect, while neglecting to cite a well-designed, earlier study which showed an impressive decrease in measles in the irradiated classrooms [17].

UVGI (UVC) stimulation of vitamin D production is not biologically plausible. Accordingly, studies in which UVGI was used for air disinfection should *not *have been biased because of augmented vitamin D production.

Lewis J. Radonovich, Jr.

North Florida/South Georgia Veterans Health System

Richard A. Martinello

VA Connecticut Healthcare System, West Haven, CT

Michael Hodgson

Veterans Health Administration

Donald K. Milton

University of Massachusetts Lowell

Edward Nardell

Harvard University

Disclaimer:

The views expressed in this manuscript are the authors' personal opinions and do not necessarily reflect the views of the authors' employers or affiliates.

## References

[B1] Cannell JJ, Zasloff M, Garland CF, Scragg R, Giovannucci E On the epidemiology of influenza. Virol J.

[B2] McLean RL (1961). The effect of ultraviolet radiation upon the transmission of epidemic influenza in long-term hospital patients. Am Rev Respir Dis.

[B3] Riley RL, Wells WF, Mills CC, Nyka, Mclean RL (1957). Air hygiene in tuberculosis: quantitative studies of infectivity and control in a pilot ward. Am Rev Tuberc.

[B4] Luckiesh M (1946). Applications of Germicidal, Erythemal, and Infrared Energy.

[B5] Wells WF (1955). Airborne Contagion and Air Hygiene: An Ecological Study of Droplet Infections.

[B6] First M, Rudnick SN, Banahan KF, Vincent RL, Brickner PW (2007). Fundamental factors affecting upper-room ultraviolet germicidal irradiation – part I. Experimental. J Occup Environ Hyg.

[B7] Rudnick SN, First MW (2007). Fundamental factors affecting upper-room ultraviolet germicidal irradiation – part II. Predicting effectiveness. J Occup Environ Hyg.

[B8] Nardell EA, Bucher SJ, Brickner PW, Wang C, Vincent RL, Becan-McBride K, James MA, Michael M, Wright JD (2008). Safety of upper-room ultraviolet germicidal air disinfection for room occupants: results from the Tuberculosis Ultraviolet Shelter Study. Public Health Rep.

[B9] First MW, Weker RA, Yasui S, Nardell EA (2005). Monitoring human exposures to upper-room germicidal ultraviolet irradiation. J Occup Environ Hyg.

[B10] Sterenborg HJ, Putte SC van der, Leun JC van der (1988). The dose-responserelationship of tumorigenesis by ultraviolet radiation of 254 nm. Photochem Photobiol.

[B11] National Institute for Occupational Safety and Health (1972). US Department of Health, Education and Welfare, Public Health service. Criteria for a recommended standard: Occupational Exposure to Ultraviolet Radiation.

[B12] First MW, Nardell EA, Chaisson W, Riley R (1999). Guidelines for the Application of Upper-Room Ultraviolet Germicidal Irradiation for Preventing Transmission of Airborne Contagion Part I: Basic Principles.

[B13] First MW, Nardell EA, Chaisson W, Riley R (1999). Guidelines for the Application of Upper-Room Ultraviolet Germicidal Irradiation for Preventing Transmission of Airborne Contagion Part II: Basic Principles.

[B14] Bruls WA, Slaper H, Leun JC van der, Berrens L (1984). Transmission of human epidermis and stratum corneum as a function of thickness in the ultraviolet and visible wavelengths. Photochem Photobiol.

[B15] MacLaughlin JA, Anderson RR, Holick MF Spectral character of sunlight modulates photosynthesis of previtamin D3 and its photoisomers in human skin. Science.

[B16] Riley RL, O'Grady F (1961). Airborne infection.

[B17] Wells WF, Wells MW, Wilder TS (1942). The environmental control of epidemic contagion I: An epidemiologic study of radiant disinfection of air in day schools. Am J Hyg.

